# Mechanisms of Apoptosis Resistance to NK Cell-Mediated Cytotoxicity in Cancer

**DOI:** 10.3390/ijms21103726

**Published:** 2020-05-25

**Authors:** Christian Sordo-Bahamonde, Seila Lorenzo-Herrero, Ángel R. Payer, Segundo Gonzalez, Alejandro López-Soto

**Affiliations:** 1 Department of Functional Biology, Immunology, University of Oviedo, 33006 Oviedo, Spain; seilalorenzoherrero@gmail.com (S.L.-H.); segundog@uniovi.es (S.G.); 2 Instituto Universitario de Oncología del Principado de Asturias, IUOPA, 33006 Oviedo, Spain; apayer.angel@gmail.com; 3 Instituto de Investigación Biosanitaria del Principado de Asturias (ISPA), 33011 Oviedo, Spain; 4 Department of Hematology, Hospital Universitario Central de Asturias (HUCA), 33011 Oviedo, Spain; 5 Department of Biochemistry and Molecular Biology, University of Oviedo, 33006 Oviedo, Spain

**Keywords:** NK cell, apoptosis, granzymes, perforin, death receptors, Trail, FasL, Fas, granulysin, apoptosis resistance

## Abstract

Natural killer (NK) cells are major contributors to immunosurveillance and control of tumor development by inducing apoptosis of malignant cells. Among the main mechanisms involved in NK cell-mediated cytotoxicity, the death receptor pathway and the release of granules containing perforin/granzymes stand out due to their efficacy in eliminating tumor cells. However, accumulated evidence suggest a profound immune suppression in the context of tumor progression affecting effector cells, such as NK cells, leading to decreased cytotoxicity. This diminished capability, together with the development of resistance to apoptosis by cancer cells, favor the loss of immunogenicity and promote immunosuppression, thus partially inducing NK cell-mediated killing resistance. Altered expression patterns of pro- and anti-apoptotic proteins along with genetic background comprise the main mechanisms of resistance to NK cell-related apoptosis. Herein, we summarize the main effector cytotoxic mechanisms against tumor cells, as well as the major resistance strategies acquired by tumor cells that hamper the extrinsic and intrinsic apoptotic pathways related to NK cell-mediated killing.

## 1. Introduction

Natural killer (NK) cells are large granular innate lymphoid cells. This immune subset is in charge of recognizing and eliminating viral-infected and tumor-transformed cells in an antigen-independent manner, playing a key role in the immune control of tumor development and metastasis [1,2,3]. Whereas cytotoxic T lymphocyte stimulation is antigen-specific dependent, NK cell activation relies on their ability to discern infected and malignantly transformed cells without any prior sensitization [1,4,5].

During NK cell ontogenesis, these innate immune cells undergo a “licensing” process whereby cells expressing major histocompatibility complex class I (MHC-I) self-specific inhibitory receptors are positively selected, acquiring functional competence. Thereby, NK cells lacking MHC-specific inhibitory receptors do not achieve functional maturation [4]. According to the missing-self hypothesis, MHC-I recognition by inhibitory receptors prevents NK cell-mediated cytotoxic activity against healthy cells. This regulation of NK cell function is mainly mediated by Killer Immunoglobulin Receptors (KIRs/CD158 family), C-type lectin receptors (CD94/NK group 2 member A, NKG2A), and leukocyte immunoglobulin-like receptor-1 (LILRB1/ILT2) [6,7].

NK cell activation is closely controlled by a precise balance between the signals provided by these inhibitory and stimulatory receptors [1]. The sum of these opposing signals determines the activation status of the NK cell, playing a crucial role in maintaining self-tolerance and limiting immune responses, which, in turn, prevents autoimmunity. NK cells recognize the increased expression of stress-induced molecules on the surface of viral-infected or cancer cells by activating receptors, such as activating receptor killer cell lectin like receptor K1 (KLRK1/NKG2D), DNAX accessory molecule 1 (DNAM-1), and the natural cytotoxicity receptors natural killer P30, 44, and 46 (NKp30, NKp44, and NKp46) [8]. In humans, NKG2D stress-induced ligands include MHC class I polypeptide-related sequence A and B (MICA and MICB) and UL16 binding proteins (ULBPs). Additionally, NK cells may also eliminate target cells which are opsonized by antibodies via engagement of Fc gamma receptor CD16 in an antibody-dependent cellular cytotoxicity (ADCC).

Upon target cell recognition, NK cells may mediate target cell lysis via direct release of lytic granules or engagement of death receptor-related apoptosis through tumor necrosis factor (TNF), Fas ligand (FasL), and TNF-related apoptosis-inducing ligand (TRAIL) production, whose surface receptors are commonly expressed by tumor cells [9]. These killing mechanisms trigger certain programmed intracellular events on target cells and constitute the biological basis of a variety of approaches employed in cancer immunotherapy, such as ADCC in the treatment of hematological malignancies (i.e., rituximab in B-cell malignancies) and solid tumors (i.e., cetuximab in head and neck cancers) [10,11,12,13,14]. However, the ultimate mechanisms that lead to tumor cell death upon exposure to immunotherapeutic treatments remains unclear. Altogether, the cytotoxic capacity of NK cells highlights the importance of this immune subset in promoting apoptosis and eliminating cancer cells. Activation of programmed cell death signaling appears to be a central player in preventing tumor progression, since resistance to apoptosis has been defined as a hallmark of cancer [15].

In this context, two main mechanisms are responsible for NK cell-mediated cytotoxicity resistance: 1) tumor cells take advantage of co-inhibitory signaling to avoid NK cell-mediated responses, leading this immune subset to an anergic or irresponsiveness state, and 2) tumor cells avoid NK cell effector activity after target cell recognition (i.e., inefficient perforin (PRF1) binding).

Further, diverse strategies conferring apoptotic resistance are present in all types of cancers, affecting both the intrinsic (via mitochondria) and extrinsic (mediated by death receptors) pathways. These include, but are not limited to, BCL2 overexpression, mutation/deletion of the *TP53* gene, as well as caspase activity dysfunction [16]. In this review, we summarize the major mechanisms affecting NK cell-mediated apoptosis and resistance in cancer.

## 2. NK Cell-Killing Mechanisms

### 2.1. NK Cell Killing Mechanisms: Death Receptors, the Extrinsic Apoptosis Pathway

The extrinsic apoptotic pathway is initiated when the so-called death ligands bind to their cognate TNF-family death receptors, promoting caspase-dependent apoptotic cell death. To date, nine different receptors have been described (Table 1).

Fas/CD95 is a membrane-bound receptor ubiquitously expressed in humans, whereas its cognate ligand (FasL/CD95L) is mainly expressed by NK cells and cytotoxic T lymphocytes [17]. Upon receptor-ligand recognition, the extrinsic apoptotic pathway is activated by a Fas death domain. This cell surface receptor modulates immune system homeostasis, limiting immune responses and promoting the elimination of malignant cells [18]. TRAIL is a type II transmembrane protein that, as with most of the TNF family members, forms homotrimers that bind to three receptor molecules [19]. TRAIL expression may be detected on immune cells from myeloid and lymphoid origin, but, interestingly, peripheral blood NK cells do not express membrane TRAIL and yet, it is constitutively expressed on liver NK cells in humans [20,21]. Nonetheless, surface expression of this molecule is upregulated on NK cells upon activation by cytokines, such as IL-15 and IFN-γ, bringing about NK cell-mediated target cell apoptosis [20].

Despite the fact that TRAIL may bind to five different receptors, only TRAIL-R1 and TRAIL-R2 are capable of inducing extrinsic apoptotic cell death through their death domain (Table 1) [22]. No death domain is present in TRAIL-R3, TRAIL-R4, or osteoprotegerin, so receptor-ligand binding does not induce caspase recruitment and activation [23]. These “decoy” receptors are in charge of protecting cells from apoptosis by competing with death receptors for TRAIL binding, activating pro-survival signaling pathways such as NF-kB (nuclear factor-κB), and forming inactive complexes with TRAIL-R2 [24,25,26,27,28].

**Figure 1:**
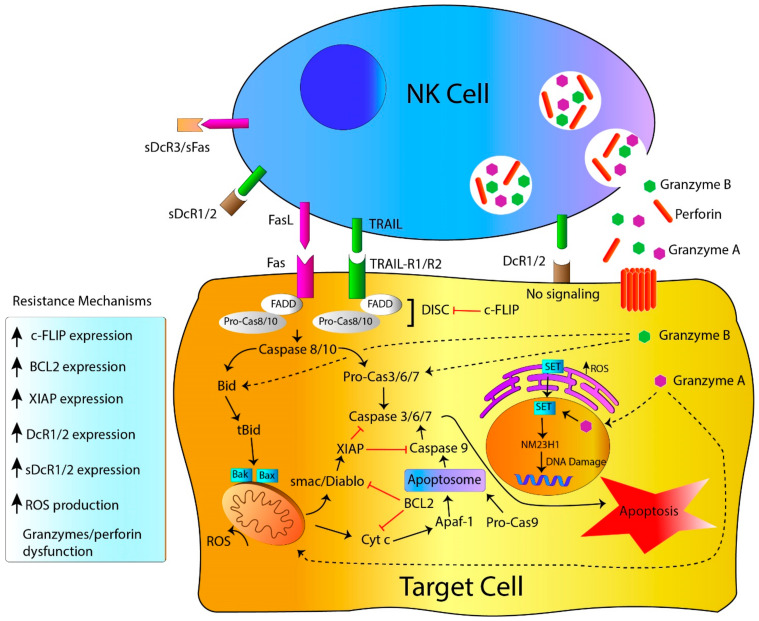
Effector mechanisms and tumor cell-resistance in NK cell-mediated apoptosis. NK cells eliminate target cells through death receptor-ligand engagement and the release of their cytotoxic granules containing granzymes and perforin. Nonetheless, genetic alterations, overexpression of anti-apoptotic proteins and the production of soluble decoy receptors represent critical factors affecting NK cell-mediated cytotoxicity.

Binding of death receptors (such as TRAIL-R1/R2 or Fas) to their respective ligands induces receptor oligomerization and, subsequently, the death domain attracts the adaptor proteins FADD (Fas-associated with death domain protein) and TRADD (TNF receptor-associated death domain) (Figure 1) [22,29]. This event initializes the recruitment of inactive proforms of procaspase-8 and procaspase-10, inducing the formation of the death-inducing signaling complex or DISC [29]. Once this complex is assembled, both procaspases are activated by cleavage, promoting the recruitment of executioner caspases (caspase-3, -6, and -7). This extrinsic signaling alone may not be enough to induce apoptosis and requires an amplification step induced by caspase-8, which targets the BH3-only protein Bid (BH3-interacting-domain death agonist), activating the intrinsic pathway [30,31]. Membrane bound forms of TRAIL and FasL exert optimal activity inducing cell death, whereas soluble forms of these proteins (cleaved by metalloproteases or cathepsin, respectively) poorly induce cell death or even exert tumor-promoting functions [32,33,34,35].

NK cells play a key role in the elimination of cancer cells and metastasis control, at least in part, due to FasL and TRAIL-mediated cell death [2,36]. Patients with autoimmune lymphoproliferative syndrome (ALPS), who bear mutations affecting FasL or caspase-10, show increased risk of Hodgkin (HL) and non-Hodgkin lymphoma (NHL), highlighting the importance of the extrinsic apoptosis pathway [37]. Further, Fas/FasL disruption in mice led to the spread of solid and hematologic tumors [38,39,40,41]. Accordingly, TRAIL-deficient mice displayed increased susceptibility to tumor development, including enhanced tumor growth in mice challenged with A20 B lymphoma cells [42]. Likewise, TRAIL^−^/^−^ mice were observed to be more sensitive to experimental and spontaneous metastasis as well as those caused by chemical carcinogens in a wide variety of cancers [43,44,45,46,47].

Altogether, these data highlight the relevance of NK cell-mediated cytotoxicity in eliminating nascent and established tumors and limiting metastasis. In parallel, cancer cells have developed numerous mechanisms counteracting the extrinsic and intrinsic apoptotic pathways or taking advantage of its mediators (see Table 2).

### 2.2. NK Cell-Killing Mechanisms: Target Cell Lysis by Perforin, Granzymes and Granulysin Granules

In addition to activation of the death receptor pathway, exocytosis of cytotoxic granules with perforin (PRF1) and granzymes (GZMs) is a major mechanism of NK cell-induced apoptotic tumor cell death. This process involves target cell recognition by the mechanisms described above (including ADCC), followed by formation of the immunological synapse via integrin-mediated adhesion, and mobilization of preformed lytic granules to the effector-target contact zone [48]. Finally, NK cells initialize the release of their cargo of preformed lytic granules containing both PRF1 and GZMs at the immunological synapse, a process known as degranulation (Figure 1) [49,50,51]. PRF1 monomers are glycoproteins that, similarly to the complement membrane attack complex, aggregate and form a pore in the membrane of the tumor cell, hence allowing the internalization of GZMs into the cytosol and eliciting osmotic imbalance [50]. Additionally, direct endocytosis of GZMs by target cells has been proposed as an alternative method to GZM protease delivery [52,53,54]. Nevertheless, *Prf1*^−^/^−^ mice demonstrated a complete abrogation of target cell death in a lytic granule-dependent manner, supporting the relevance of the pore formation over the direct endocytosis of GZMs [55,56]. Furthermore, PRF1 induces the formation of target cell membrane invaginations, allowing GZMs to be delivered by endocytosis [52].

PRF1 is essential for the NK cell control of tumor development and metastasis [57,58,59,60]. Indeed, PRF1-deficient mice developed B-cell lymphomas in 60% of cases [58]. Accordingly, patients with type 2 familial hemophagocytic lymphohistiocytosis with biallelic mutations affecting the *PRF1* gene were observed to develop hematological malignancies in 50% of cases during childhood or adolescence, whereas NK cell dysfunction has been described [61,62]. 

GZMs are proteins from the family of serine proteases that are localized in granules, preventing the host cell from being damaged by its own cargo. To date, five different GZMs have been described in humans (A, B, H, K, and M). Granzymes A (GZMA) and B (GZMB) are the most abundant constituents of granules and have been deeply studied due to its important function in eliminating malignant or transforming cells. On the contrary, the role of GZMH, -K, and -M remains poorly understood [127,128,129].

GZMA induces cell death by a caspase-independent pathway. This serine protease alters the mitochondrial inner membrane potential, leading to the release of reactive oxygen species (ROS) and, in contrast to GZMB, does not affect pro-apoptotic proteins such as smac/DIABLO or cytochrome c [130]. ROS production prompts endoplasmic reticulum (ER)-associated SET complex translocation to the nucleus, where SET is cleaved by GZMA, releasing nucleases associated to DNA damage, such as NM23-H1 DNase that, along with TRX1, degrades DNA and leads to cell death [131,132,133]. This protease also targets histone H1, KU70, and DNA damage sensor poly(adenosine 5′-diphosphate-ribose) polymerase-1 (PARP), presumably facilitating DNase activity [134,135]. Likewise, GZMA has a trypsin-like activity, cleaving after asparagine (Asp) and lysine (Lys) residues, which generates single-stranded DNA nicks that cannot be detected in GZMB-related apoptosis [136].

GZMB is generally expressed by NK cells and cytotoxic T lymphocytes, although this protease may also be found in myeloid cells, such as macrophages, plasmacytoid dendritic cells, neutrophils, basophils, or mast cells [137,138,139,140]. This serine protease essentially cleaves after Asp residues, showing a wide variety of substrates. As an example, GZMB exerts a direct proteolytic processing of executioner procaspases (being its main targets caspase-3 and -7), hence eliciting caspase-dependent apoptosis [141]. GZMB also cleaves the BH3-only protein Bid, inducing the truncated form of this protein and leading to Bak/Bax activation and pore formation on the mitochondria outer membrane, followed by the release of pro-apoptotic proteins, such as smac/DIABLO, cytochrome c, high temperature requirement A2 (HtrA2)/Omi serine protease, apoptosis inducing factor (AIF), and endonuclease-G (Endo-G) [141].

Granzyme M (GZMM) is abundantly expressed on NK cells and has been classically related to innate immune responses. This granzyme promotes caspase- and mitochondria-independent cell death by direct cleavage of α-tubulin and actin-plasma membrane linker ezrin, targeting key components of the cytoskeleton [142,143]. To date, the role of GZMM in immunosurveillance and its antitumor activity have not been fully clarified. On the one hand, GZMM-deficient mice displayed normal NK cell development and cytotoxic capacity, suggesting that this granzyme does not play a crucial role on NK cell-mediated cytotoxicity [144]. Further, GZZM has been described to promote epithelial-to-mesenchymal-transition (EMT) in vitro [145]. On the other hand, the generation of GZMB^−^/^−^ and GZMM^−^/^−^ mice models unveiled that both granzymes are required for growth inhibition of a transplanted sarcoma cell line during adoptive NK cell transfer [146]. Further, GZMM has been demonstrated to effectively inactivate proteinase inhibitor 9, a GZMB inhibitor, suggesting that the former may indirectly play an antitumor role [147].

Although, as mentioned above, deficiencies in PRF1 correlate with diminished target cell lysis by effector T lymphocytes and NK cells and increased risk of cancer, the importance of lacking individual GZMs remains elusive [148,149]. Mice deficient in GZMA and GZMB have been reported to show an increased sensitivity to NK cell-mediated cytotoxicity [150,151]. Interestingly, GZMB expression has been reported in cancer cells from diverse solid tumors, such as breast cancer, head and neck cancer, and lung carcinoma, where it has been suggested to intervene in extracellular matrix remodeling, promoting EMT [151,152,153]. In line with this, GZMB expression has been suggested to promote tumor immunoevasion, favoring B regulatory (Breg) and T regulatory (Treg)-mediated immune suppression, dampening antitumor NK cell and T lymphocyte responses, albeit contradictory results that have been obtained to date [154,155,156,157,158].

Granulysin is delivered, along with GZMs, in a PRF1-dependent manner. Granulysin is a saposin-like antibacterial cytolytic protein expressed by NK cells and cytotoxic T lymphocytes. It is mainly involved in host defense against intracellular pathogens by altering the target cell membrane permeability. In the context of cancer, granulysin-mediated apoptosis may be caspase-dependent or independent [159]. Granulysin is able to activate plasma membrane sphingomyelinase, triggering sphingomyelin degradation and ceramide production, and stimulating pro-apoptotic pathways [160]. Likewise, this cytolytic protein may alter ER membrane permeability, increasing the intracellular levels of cytosolic Ca^2+^, ROS production, and inducing the loss of mitochondrial membrane potential [161,162]. Altogether, this intracellular cascade of events activates caspase-dependent apoptosis, via cytochrome c release among other pro-apoptotic factors [162,163,164]. However, little evidence of the role of granulysin in antitumor immunity are available, since in vivo activity has not been completely demonstrated. Increased granulysin expression has been correlated to good prognosis in a wide variety of cancers, including solid and hematological malignancies [165,166,167,168,169,170]. Therapeutic strategies based on granulysin analogues have been studied, showing in vitro activity in cell lines and primary chronic lymphocytic leukemia (CLL) and multiple myeloma (MM) cells [171]. However, scarce data about its potential in vivo are available to date and deserves further investigations [172,173].

## 3. Resistance to NK Cell-Mediated Cytotoxicity

### 3.1. Resistance to Apoptosis: Genetic Alterations Linked to Genes Governing Apoptosis

Tumor cells undergo a wide number of genetic and epigenetic alterations throughout cancer development that provide survival advantages, including the ability to evade apoptosis [15]. Concomitantly, malignant cells become more resistant to NK cell-mediated elimination, escaping immune surveillance and, hence, favoring tumor progression. Tumor-derived alterations conferring resistance to programmed cell death usually affect genes controlling apoptotic signaling pathways at different levels. A common mechanism to suppress apoptosis in tumor cells relies on the impairment of caspase function, frequently via genetic modifications. For instance, caspase-8 is highly mutated in human cancers [174]. An early screening study identified thirteen different mutations in the *CASP8* gene, which altered protein activation, in an array of tumors from diverse origins, with a higher incidence of mutation in gastric cancer [63]. Further, a caspase-8-defective mutant characterized by a single amino acid deletion (Leu62del) was described in vulvar squamous carcinoma and a frameshift mutation (1225_1226delTG) is likely responsible for the loss of caspase-8 activity in hepatocellular carcinoma, suggesting that genetic alterations linked to caspase-8 inactivation might vary depending on the type of cancer [64,65]. A recent work regarding *CASP6*, a significantly mutated gene in colon and gastric cancer [66], found tumor-associated mutations that decrease the catalytic efficiency of the protein, thus contributing to the resistance of cancer cells to programmed cell death [175]. Likewise, analysis of *CASP3* gene sequence revealed fourteen somatic mutations throughout a panel of tumor samples from different origins, alterations that might render this protein insensitive to GZMB-mediated cleavage and, thus, directly interfere with NK cell-mediated killing [67]. Several other studies also unveiled the presence of inactivating mutations in tumor cells that hinder the function of other caspases, such as caspase-4, -5, -7, and -9 [68,69,70,71].

Tumor cells may also become resistant to programmed cell death through the abnormal activity of molecules that suppress the apoptotic cascade. At this respect, the aberrant expression of survivin is achieved in advanced stages of neuroblastoma via chromosome 17q gain, as occurs with c-IAP1, a protein overexpressed in a wide variety of tumors due to gene amplification [72,73,74]. Alternatively, c-IAP2 shows enhanced activity as a consequence of chromosomal translocations. The gene rearrangement (t(11;18)) results in the fusion protein c-IAP2/MALT1, present in a large proportion of MALT (mucosa-associated lymphoid tissue) lymphomas [75]. This protein might confer resistance to apoptosis through interaction with regulators of the apoptotic program, such as smac/DIABLO or TRAF2 (TNF receptor-associated factor 2) [75,176,177].

Additionally, downregulation or suppression of pro-apoptotic proteins by tumor cells results in resistance to NK cell-mediated apoptosis. The large group of proteins that regulate the mitochondrial apoptotic response is subjected to genetic modulation as well. Frameshift mutations leading to inactivation of the pro-apoptotic protein Bax have extensively been described in colon and gastric cancers with microsatellite instability [76,77,78]. These tumors also harbor mutations in *Apaf-1* in a low frequency, which might contribute to cancer progression via impairment of a correct assembly of the apoptosome. *BAX* gene mutations have also been identified in certain hematologic malignancies, such as CLL or Burkitt’s lymphoma [79,80,178]. In line with this, the *BCL2* locus is subjected to chromosomal translocation (t(14;18)), a typical feature reported in diffuse large B-cell lymphoma (DLBCL) that translates into BCL2 overexpression [82]. Likewise, oncogenic viruses, such as human herpesvirus 8 (HHV8), encode homologue proteins to BCL2 and FLICE (FADD-like IL-1β-converting enzyme)-inhibitory protein (c-FLIP), therefore protecting cancer cells from apoptosis [83,84]. Other members of the BCL2 family of proteins also carry tumor-associated alterations that tip the intracellular balance towards an anti-apoptotic state, e.g., Bim deletion in mantle cell lymphoma (MCL) or Noxa mutations in DLBCL [88,89]. Overexpression of BCL2 family members has also been associated with ADCC-based therapy resistance [179,180].

In general, the alterations described herein ultimately result in attenuation or loss of the apoptotic potential of tumor cells, a key stage in NK cell-mediated elimination. Hence, cancer-associated alterations that lead to functionally defective apoptosis not only provide survival advantages in terms of tumor development and progression, but also evade the immune surveillance carried out by NK cells and other cytotoxic immune subsets. The particular features of tumor cells that provide protection against PRF1/GZMs and death receptor-mediated NK cell cytotoxicity will be discussed next.

### 3.2. Resistance to Death Receptors-Mediated Cell Death

As previously described, the activation of the extrinsic apoptotic cascade relies on the engagement of death receptors that transduce the death signal to intracellular components of the pathway. Consequently, inactivation of such receptors results in the dysregulation of apoptosis, a strategy linked to tumor progression. A total of seven mutations in TRAIL receptors (three in *TRAIL-R1* and four in *TRAIL-R2*) were detected in metastatic breast cancer samples and the expression of each mutant variant translated into defective apoptosis in HEK293 cells [93]. Somatic mutations in TRAIL receptor genes have also been described in various human tumors, although their frequency is relatively low [90,91,95,96]. Four of the tumor-associated mutations identified so far (L334F, E326K, E338K, and K386N) caused loss of TRAIL-R2 function via defective recruitment of intracellular components, including caspase-8 or FADD [181]. Further, the loss of chromosome 8p correlated with downregulation of TRAIL-R1 and TRAIL-R2 and, concomitantly, resistance to TRAIL-induced apoptosis in an array of B-cell malignancies [92]. This allelic deletion is typically present in epithelial carcinomas as well and is associated with poor prognosis and metastasis [93,182,183,184,185]. Following the same line, *FAS* gene exhibits loss-of-function mutations in tumor cells that avoid apoptosis activation upon CD95L/FasL engagement [97].

As mentioned above, NK cells eliminate target cells by the activation of death receptors and release of cytotoxic granules containing PRF1, GZMs, and granulysin. Nevertheless, both mechanisms rely on the tumor cell apoptotic machinery. Indeed, the alteration of apoptosis mediators are present in all types of tumors, representing a critical hallmark of cancer [15].

The DISC complex function upon receptor-ligand binding is tightly regulated by c-FLIP, a homologue of caspase-8 that lacks caspase activity and binds to FADD and caspase-8 or -10, thereby inhibiting the DISC complex formation. High expression of c-FLIP, countervailing TRAIL-mediated apoptosis, has been correlated with apoptosis resistance and poor prognosis in a wide variety of solid tumors and hematological malignancies [85,86,87,186,187]. Further, c-FLIP overexpression is related to extrinsic apoptosis, but not to GZM/PRF1 pathway resistance, whereas its role in chemotherapy-mediated apoptosis resilience remains elusive [188,189,190,191]. In vivo, c-FLIP overexpression protected tumor cells from lysis by NK cells in a PRF1-deficient murine model, once again highlighting the importance of this protein in cancer [192]. As a result of alternative splicing, three different c-FLIP variants are expressed: c-FLIP-long (c-FLIP_L_), c-FLIP-short (c-FLIP_S_), and c-FLIP-Raji (c-FLIP_R_). All three variants conserve the death effector domain (DED) that binds to the DISC complex, hence inhibiting apoptosis [193,194]. Furthermore, c-FLIP_L_ acts as an anti-apoptotic protein depending on its expression level; high levels of c-FLIP_L_ compete with caspase-8 showing anti-apoptotic features, whereas low levels of c-FLIP_L_ enhance procaspase-8 processing, thus favoring apoptosis [195].

Overexpression of decoy receptors stands as a resistance mechanism linked to death receptors, since it has been correlated to poor prognosis and lack of response to FasL and TRAIL. Decoy receptor 3 (DcR3) is a protein related to FasL and LIGHT (TNFSF14) suppression, whose aberrant expression has been detected in several tumors and might play a role in triggering EMT [100,101,102,196,197]. Consistently, TRAIL-R3 and TRAIL-R4 altered expression has been described in several tumors, including breast, prostate, and pancreatic cancers [103,104,105,198,199]. Likewise, the anti-apoptotic activity of decoy receptors not only relies on receptor-ligand competition; TRAIL-R4 activates and sustains NF-κB signaling, thus promoting leukemic cell survival [200,201]. In line with this, the expression of soluble receptors that behave as a decoy for death ligands represents another resistance mechanism associated with the extrinsic apoptotic pathway [98,99]. Besides the pro-apoptotic and anti-tumor functions of death receptors, tumor-promoting functions have also been described for Fas/FasL. Growing evidence, such as Fas expression on cancer cells, suggest that this protein also harbors non-apoptotic activity. Furthermore, elimination of Fas/FasL in mouse models of ovarian and liver cancers resulted in reduced tumor incidence and growth, probably due to the death induced by Fas/FasL elimination (DICE) phenomenon [202]. Further, triggering DICE in cancer cells by targeting the Fas/FasL axis might represent a therapeutic option in the near future [203,204].

Although in vitro/in vivo and preclinical TRAIL-based therapies demonstrated encouraging results and low toxicity owing to tumor-specific activity, the use of death receptors, such as FasL or TNF, as anticancer therapy was soon dampened [205,206,207,208,209,210]. Different approaches to TRAIL-based therapies have been suggested, including recombinant TRAIL and agonistic monoclonal antibodies (mAbs), targeting TRAIL-R1 and/or TRAIL-R2. Despite that fact that TRAIL-based therapies were generally well tolerated, phase I/II clinical trials for a wide variety of cancers resulted in poor responses for the vast majority of patients [211,212,213,214,215,216]. Withal, different resistance mechanisms seem to limit the success of TRAIL-based therapies: (i) low persistence of recombinant TRAIL, (ii) whereas agonistics antibodies are receptor specific, recombinant TRAIL also binds to decoy receptors, (iii) depending on the tumor, TRAIL receptor apoptotic signaling preferably relies on TRAIL-R1 or TRAIL-R2, so agonistic mAbs to specific receptors must be carefully chosen [217,218,219,220], (iv) agonistic mAbs are weak apoptosis-inducers due the lack of death receptor homotrimers (reviewed in [21] and [221]) and, (v) in some cases, severe hepatotoxicity was detected [222,223,224,225]. Due to the lack of success of recombinant TRAIL and agonistic mAbs as monotherapy, combinatorial therapies along with CDK9 inhibitors or SMAC mimetics among others have currently been studied [226,227,228,229,230].

### 3.3. Resistance to Perforin, Granzymes and Granulysin-Mediated Cell Death

Tumor cells have evolved distinct strategies to interfere with PRF1 and/or GZM function, thus leading to immune escape. Such NK cell shaping may be achieved via direct mechanisms, such as release of soluble factors by cancer cells or through recruitment of suppressor cells that indirectly hinder NK cell antitumor activity. Since ADCC-mediated cytotoxicity is PRF1 and GZM-dependent, resistance mechanisms targeting these immune mediators limit the efficacy of ADCC-based therapies [231,232]. Further, loss of targeted antigens or antibody endocytic uptake also correlate with faulty ADCC [233].

Given the largely proven vital role of PRF1 in NK cell-mediated tumor rejection, this protein arises as a highly-targeted component of the degranulation pathway [57,58,59]. A typical strategy reported in a wide variety of tumors entails the inhibition or downregulation of PRF1 expression on NK cells [106,107,108,124,234]. In line with this, coculture experiments unveiled that myeloid-derived suppressor cells (MDSCs) from mammary adenocarcinoma-bearing mice reduced PRF1 levels in NK cells, correlating with decreased NK cell cytotoxicity in vivo [109]. Cancer-associated fibroblasts (CAFs) also display a suppressive activity that partially relies on PRF1 downmodulation in NK cells [110,111,112]. This mechanism of NK cell evasion not only affects PRF1 expression; several studies have reported lower levels of GZMB, as well as, CD107a in tumor-infiltrating NK cells, an altered phenotype that translates into an attenuated antitumor immune response [107,108,115,124,235]. Apart from this extended evasion strategy, an early study involving acute myeloid leukemia (AML) samples revealed that the intrinsic apoptosis resistance associated with this type of cancer relies on an impaired binding of PRF1 to the surface of the tumor cells [113]. Similarly, TGF-β-producing tumors are more likely to resist NK cell-mediated cytotoxicity, since PRF1 mobilization to the immune synapse was abrogated in NK cells cocultured with Burkitt’s lymphoma Raji cells in the presence of this immunosuppressive cytokine [114]. A recent study in breast cancer cell lines discovered a substantial accumulation of F-actin near the immune synapse in tumor cells upon encountering NK cells [125]. Inhibition of this actin response led to an increase in NK cell-mediated apoptosis of tumor cells, hence bringing to light a novel mechanism of immune escape. The stability of the immune synapse is crucial for an efficient cytolytic killing of tumor cells upon NK cell recognition. Hypoxic conditions, through activation of the transcription factor HIF-1α, triggered selective degradation of connexin-43, a major component of gap junctions in melanoma cells, which correlated with decreased susceptibility to NK cell cytotoxicity due to immune synapse destabilization [126]. The adverse tumor microenvironment may therefore interfere with the degranulation process, actively contributing to tumor resistance to NK cell-mediated apoptosis, which will be further discussed in this work. Accordingly, GZMB degradation was boosted by activation of autophagy in hypoxic human breast cancer cells [116,117]. Inhibition of autophagy by targeting beclin1, a key regulator of autophagosome formation, restored the killing capacity of NK cells in vivo, together with the presence of GZMB in hypoxic tumor cells in vitro. Serine protease inhibitors, best known as serpins, stand out as a major culprit of resistance to apoptosis in tumor cells, since they are suppressors of different members of the GZM family. Serpin B9 (protease inhibitor 9; PI-9) specifically targets the proteolytic activity of GZMB and diverse studies have reported increased intracellular expression of this serpin in a broad subset of human cancers [118,119,120,236,237]. Serpin B9-expressing tumor cell lines proved to be more resistant to GZMB-induced apoptosis, implying that this evasion strategy might weaken the tumoricidal potential of NK cells via blockade of the degranulation pathway [237]. Along this line, in vitro experiments revealed serpin B4 (squamous cell carcinoma antigen 2; SCCA-2) as an inhibitor of GZMM-mediated cell death, which is highly expressed by squamous cell carcinomas [121,122,123,238]. NK cell-induced apoptosis was reduced upon overexpression of serpin B4 in HeLa cells, further reinforcing the role of serpins in reducing tumor elimination by the immune system.

No direct impairment of granulysin function has been described in cancer so far. An early report established a link between lower expression of granulysin on NK cells and cancer progression in patients with disparate types of cancer [168]. Additionally, this decreased granulysin expression correlated with reduced numbers of circulating NK cells, which might further contribute to tumorigenesis. A set of mechanisms acquired by tumor cells that negatively target granulysin expression, hence attenuating the antitumor potential of NK cells, might explain these observations. Nonetheless, the relevance of defective or decreased granulysin levels in tumor evasion to immune elimination merits future investigation.

Altogether, these data bring to light the importance of the PRF1/GZM pathway in cancer immunosurveillance, since tumor cells have developed a myriad of mechanisms to interfere with this process, acquiring resistance to NK cell-mediated apoptosis. Restoring this degranulation capacity might prove a useful immunotherapeutic approach to achieve tumor rejection in patients with cancer.

### 3.4. Resistance to Apoptosis: Influence of the Tumor Microenvironment

The diverse components—from tumor-associated immune cells to stromal cells or even soluble factors—of the tumor microenvironment (TME) take an active part in cancer establishment and progression at different levels. Tumor cells exploit the adverse conditions of the TME for their own benefit, for instance, by avoiding NK cell-mediated recognition and cytotoxicity or favoring immunosuppression.

The TME typically displays chronic stress conditions, such as hypoxia or oxidative stress, that can negatively affect NK cell antitumor function both in a direct manner or via other cell subsets [5]. At this respect, the presence of ROS in the TME frequently translates into NK cell dysfunction [239,240]. In vitro studies reported a decrease in NKG2D and NKp46 levels in CD56^dim^ NK cells elicited by phagocyte-derived ROS, which might account for the oxidative stress-associated attenuation of NK cell cytotoxicity [241]. Likewise, hypoxia led to downregulation of activating receptors on NK cells in vitro, which correlated with reduced tumor sensitivity to NK cell-mediated elimination [242]. The detrimental effect of hypoxia on NK cells has also been described in in vivo settings, reinforcing the role of stress conditions in tumor evasion of NK cell responses [243,244].

Upon exposure to hypoxic stress, tumor cells recruit immunosuppressive cells to the TME via chemotactic factors. As an illustration, MDSCs arrive to the tumor niche following the trail of tumor-derived CCL26 (C–C motif chemokine ligand 26) in hepatocellular carcinoma [245]. MDSCs, as already mentioned, suppress NK cell function in the TME by inhibiting perforin production, among other mechanisms [109]. Treg cells and tumor-associated macrophages (TAMs) infiltrate the TME, where, together with MDSCs, they release immunosuppressive cytokines, mainly TGF-β, which is largely known to hamper NK cell-mediated tumor killing, including ADCC, by decreasing *GZMB* and *FasL* expression in NK cells [14,246,247,248,249,250,251,252]. Further, ROS production by these subsets strengthens the oxidative status of the TME, hence deepening NK cell suppression [239,253].

Additionally, tumor cells undergo distinct adaptations to overcome the challenging conditions of the TME, some of which confer apoptotic resistance. Hypoxia modulates the balance of crucial proteins governing apoptosis, tipping the scale towards an anti-apoptotic cellular state. Hypoxic tumor cells display reduced levels of pro-apoptotic members of the BCL2 family, such as Bax [254,255]. Conversely, several anti-apoptotic proteins are increased in hypoxic tumor cells, including c-IAP2 and Mcl-1 [256,257]. Similarly, ROS-dependent stimulation of Akt might lead to apoptosis evasion through inactivation of caspase-9 and Bad [258,259]. These survival advantages acquired by tumor cells under stress situations not only support cancer progression, but also protect malignant cells from NK cell-mediated cell death.

Interestingly, non-immune cells from the tumor site contribute to cancer resistance to NK cell-mediated apoptosis. The tumor-associated stroma is predominantly represented by CAFs in the majority of cancers [260]. These stromal cells shape the TME and are also strongly influenced by other elements present in the tumor site. Recent studies brought to light an inhibitory effect of colon carcinoma-isolated CAFs on NK cell activity, which partly relied on the downregulation of NKG2D and NKp44 as well as perforin and GZMB expression [112,261]. In a similar manner, melanoma-associated fibroblasts exhibited increased secretion of matrix metalloproteinases (MMPs) that, in turn, release soluble MICA and MICB from tumor cells, reducing the surface levels of these NK cell activating ligands and favoring NK cell exhaustion [8,262]. CAFs also secrete a whole array of immunosuppressive cytokines that limit immune responses, such as IL-6, IL-10, and TGF-β [263,264,265]. Among these molecules, prostaglandin E2 (PGE2) and indoleamine 2,3-dioxygenase (IDO) stand out as central regulators of NK cell function. NK cell killing of tumor cells was compromised as a result of PGE2 and/or IDO production by CAFs in several types of cancer like melanoma, hepatocellular carcinoma, and thyroid cancer [110,111,266,267]. Mesenchymal stem cells, another stromal cell population, have also been described to attenuate NK cell tumor elimination via PGE2 and IDO secretion to the TME, further supporting the role of the tumor stroma in the evasion of cancer surveillance exerted by NK cells [268,269].

The harsh conditions of the TME together with the cellular components of the tumor niche deeply contribute to creating an immunosuppressive environment that restricts NK cell activity, promoting tumor progression and resistance to apoptotic cell death.

## 4. Concluding Remarks

The antitumor function of the immune system strongly relies in the induction of apoptotic malignant cell death by certain cytotoxic lymphocytes. Among these populations, NK cells stand out as cells with a superior tumor cell killing activity via exocytosis of granules bearing pro-apoptotic mediators or activation of the death receptor pathway. However, malignant cells often subvert NK cell function by manifold mechanisms, which we outlined in the review herein. Most (if not all) anticancer chemotherapies are linked to tumor cell demise brought about by induction of, mostly, mitochondrial pathway-dependent programmed cell death. While malignant cell resilience to conventional therapeutic interventions is frequently observed, the immune surveillance of tumors operates as an extrinsic control mechanism; alerted upon cancer cell overexpression of stress-regulated self-molecules, NK cells trigger target cell death via promotion of apoptosis. Consequently, malignant cells endowed with mechanisms that circumvent apoptosis might be resilient to both certain therapies and the cytotoxic response elicited by NK cells. Hence, a great deal of effort needs to be put in order to unveil hitherto unknown cell intrinsic and extrinsic mechanisms that render tumor cells resistant to therapeutic and immune-dependent induction of apoptosis. In this sense, several combination therapies employing targeted agents and immunostimulatory mediators, which are currently under clinical evaluation, are promising regimens to overcome cancer cell resilience, limiting malignant progression by inducing apoptosis in, otherwise resistant, cancer cells.

## Figures and Tables

**Table 1:** Death receptors and ligands.

Receptor Name	Alternative Name	Ligands	Death Domains
TNF-R1	P55/P60	TNF-α	Y
Fas	CD95/APO-1	FasL (CD95L)	Y
TRAIL-R1	DR4	TRAIL	Y
TRAIL-R2	DR5	TRAIL	Y
TRAIL-R3	DcR1	TRAIL	N
TRAIL-R4	DcR2	TRAIL	N
Osteoprotegerin	OPG	TRAIL	N
EDAR	Ectodysplasin-A receptor	Ectodysplasin A (EDA)	N
NGFR	P75(NTR)	NGF, BDNF, NTF3, NTF4	Y

Y = yes; N = no.

**Table 2:** Mechanisms of resistance to NK cell-mediated cytotoxicity.

Protein	Mechanism of Resistance	Cancer	Reference
**Caspase-3**	Gene mutations that interfere with caspase activity	Gastric carcinoma	[63]
		Vulvar squamous carcinoma	[64]
		Hepatocellular carcinoma	[65]
**Caspase-4**		Advanced gastric adenocarcinoma and colorectal cancer	[66]
**Caspase-5**		MM, NHL, NSCLC, hepatocellular, colorectal and gastric carcinomas	[67]
**Caspase-6**		GIST	[68]
**Caspase-7**		NSCLC, colorectal and gastric carcinomas	[69]
**Caspase-8**		Colorectal, esophageal and head and neck carcinomas	[70]
**Caspase-9**		Colorectal and gastric carcinomas	[71]
**Survivin**	Aberrant expression by chromosomal amplification	Neuroblastoma	[72]
**c-IAP1**		Esophageal, liver, lung and ovarian carcinomas	[73,74]
**c-IAP2**	Aberrant activity by chromosomal translocation	MALT lymphomas	[75]
**Bax**	Inactivating gene mutations	Colon and gastric carcinomas with microsatellite instability	[76,77,78]
		T-ALL	[79]
		CLL	[80]
		Burkitt’s lymphoma	[81]
**BCL2**	Aberrant expression by chromosomal translocation	DLBCL	[82]
	Homologue protein expression by cancer-associated viruses	Kaposi’s sarcoma	[83]
**c-FLIP**	Homologue protein expression by cancer-associated viruses	Kaposi’s sarcoma	[84]
	Aberrant protein expression	Burkitt’s lymphoma	[85]
		AML	[86]
		Colorectal cancer	[87]
**Bim**	Gene deletion	MCL	[88,89]
**Noxa**	Silencing gene mutations	DLBCL	[89]
**TRAIL-R1**	Gene mutations that interfere with receptor activity	Lung, head and neck and gastric carcinomas	[90]
		NHL	[91]
	Allelic deletion	B-NHL	[92]
		Breast cancer	[93]
**TRAIL-R2**	Allelic deletion	B-NHL	[92]
		Breast cancer	[93]
	Loss-of-function mutations	Head and neck and lung carcinomas	[94]
		NSCLC	[95]
		Gastric cancer	[96]
	Gene mutations that interfere with receptor activity	NHL	[91]
**Fas**	Loss-of-function mutations	Hematological malignancies	[97]
**Osteoprotegerin**	Aberrant protein expression	MM	[98]
**DcR3**		Glioblastoma	[99]
		Breast cancer	[100]
		Gastric cancer	[101]
		Colorectal cancer	[102]
**TRAIL-R3**		AML	[103]
		Breast cancer	[104]
**TRAIL-R4**		Prostate cancer	[105]
**PRF1**	Downregulation of protein expression	Pancreatic, gastric and colorectal carcinomas	[106]
		Lung cancer	[107]
		Hepatocellular carcinoma	[108]
	Reduction of protein levels by tumor-associated cells	T-cell lymphoma	[109]
		Hepatocellular carcinoma	[110]
		Melanoma	[111]
		Colorectal cancer	[112]
	Impaired cell surface binding	AML	[113]
	Impaired protein mobilization to the immune synapse	Burkitt’s lymphoma	[114]
**GMZB**	Downregulation of protein expression	Lung cancer	[115]
		Hepatocellular carcinoma	[108]
		Lung cancer	[107]
	Increased protein degradation	Breast cancer	[116,117]
**Serpin B9**	Aberrant protein expression	DLBCL	[118]
		NSCLC	[119]
		Lung cancer	[120]
**Serpin B4**		Squamous cell carcinomas	[121,122,123]
**CD107a**	Downregulation of protein expression	Pancreatic cancer	[124]
**F-actin**	Intracellular accumulation	Breast cancer	[125]
**Connexin-43**	Increased protein degradation	Melanoma	[126]

NSCLC = non-small cell lung cancer, GIST = gastrointestinal stromal tumor, MALT = mucosa-associated lymphoid tissue, CLL = chronic lymphocytic leukemia, ALL = acute lymphoid leukemia, DLBCL = diffuse large B-cell lymphoma, AML = acute myeloid leukemia, MCL = mantle cell lymphoma, MM = Multiple myeloma, NHL = non-Hodgkin lymphoma.
